# Application of Vertical Electrodes in Microfluidic Channels for Impedance Analysis

**DOI:** 10.3390/mi7060096

**Published:** 2016-05-25

**Authors:** Qiang Li, Yong J. Yuan

**Affiliations:** Laboratory of Biosensing and MicroMechatronics, School of Materials Science and Engineering, Southwest Jiaotong University, 610031 Chengdu, China; liqiang811054715@163.com

**Keywords:** microfluidic, vertical electrodes, impedance, SU-8-PDMS bonding

## Abstract

This paper presents a microfluidic device with electroplated vertical electrodes in the side walls for impedance measurement. Based on the proposed device, the impedance of NaCl solutions with different concentrations and polystyrene microspheres with different sizes was measured and analyzed. The electroplating and SU-8-PDMS (SU-8-poly(dimethylsiloxane)) bonding technologies were firstly integrated for the fabrication of the proposed microfluidic device, resulting in a tightly three-dimensional structure for practical application. The magnitude of impedance of the tested solutions in the frequency range of 1 Hz to 100 kHz was analyzed by the Zennium electrochemical workstation. The results show that the newly designed microfluidic device has potential for impedance analysis with the advantages of ease of fabrication and the integration of 3D electrodes in the side walls. The newly designed impedance sensor can distinguish different concentrations of polystyrene microspheres and may have potential for cell counting in biological areas. By integrating with other techniques such as dielectrophoresis (DEP) and biological recognition technology, the proposed device may have potential for the assay to identify foodborne pathogen bacteria.

## 1. Introduction

Microfluidic devices, such as lab-on-chip or micro-total analysis systems (µTAS), have drawn significant attention in various fields for chemical synthesis [1,2], biological analyses [3,4] and environmental monitoring [5,6]. Microfluidic devices generally integrate the functions of fluid flow control, mixing, filtration and reaction. The detection of reaction extent and reaction product plays an important role in microfluidic devices, since accuracy control of chemical reactions is required nowadays. Due to low cost, low power, and ease of integrating with microfluidic devices, impedance biosensors have been applied in many fields of science and technology [7]. For instance, Iliescu *et al.* developed a microfluidic device for specific identification and characterization of cells in suspensions based on impedance measurements in 2007 [8]. Sabuncu *et al.* reported on a microfluidic impedance device for cell diagnosis in 2012 [9]. In addition, DNA sequences and any particular protein can be detected or quantified by the impedance biosensor [10,11]. Miniaturization of the impedance measurement into biochips has been proven to be very successful in maximizing the impedance signal, increasing sensitivity, and reducing detection time for the assay of foodborne pathogenic bacteria [12]. Furthermore, the integration of impedance with biological identification and concentration techniques for the selective capture of target bacteria cells has led to the development of impedance biosensors [13,14]. As a concentration method, dielectrophoresis (DEP), which allows the manipulation of the biological cells in a liquid suspension, has been widely used in impedance measurement [15,16]. The assay time for bacterial detection is reduced since the process of enriching the bacterial population by conventional cell culture methods has been eliminated. In addition, to specifically capture cells in suspension, an antigen-antibody reaction was integrated into the impedance device by immobilizing specific antibodies on the electrode surface [17,18]. When cells attach on an electrode surface, it alters the interface impedance. Due to the insulating properties of the cell membrane, it results in an increase in the interface impedance [19]. Compared with the complexity and relatively high cost of the optical method for the detection and quantification of samples, the impedance measurement is simple and can be applied to point-of-care applications [20]. In addition, real-time detection can be implemented based on the quick response of the impedance biosensor. The integration of impedance measurement and the microfluidic device provides the advantages of low cost and rapid, sensitive and selective detection [21]. Since the detection of foodborne pathogens *Salmonella*, *Escherichia coli*, *Shigella*, and *Listeria monocytogenes* in seafood by the polymerase chain reaction (PCR) method is time-consuming, the advantage of impedance detection provides an alternative way to realize real-time detection. 

The bonding method for SU-8 and PDMS has been widely reported and presented in the literature, including lamination [22] and surface modification [23]. The lamination method reported by Wang *et al.* may cause the problem of blockage since a soft, thin SU-8 layer which was partially cured was used as an intermediate layer for adhesion [24]. Surface modification was considered in this study by taking the advantages of simple operation, no blockage and strong irreversible bonding. Due to the stress concentration problem in the SU-8 layer when temperature change is experienced in the fabrication process, a low temperature method to irreversibly bond SU-8 and PDMS is preferred. Furthermore, low temperature (<90 °C) bonding methods are required, especially in cases where the devices to be sealed contain biological agents or pre-dispensed chemicals. A low temperature surface modification for bonding between SU-8 and PDMS was reported by Vlachopoulou *et al.* [25], Zhang *et al.* [23], and Talaei *et al.* [26]. By introducing Si-containing groups on the surface of the SU-8 layer through 3-APTES (3-aminopropyltriethoxysilane)-functionalization, the amino group was immobilized on the surface to be bonded [25]. With the subsequent oxygen plasma treatment, the surface-functionalized SU-8 layer was irreversibly bonded to the oxygen plasma–treated PDMS layer, resulting in strong and homogeneous bonding. With the advantage of being comparatively simple to implement and cheap to perform, this method is selected to be used for SU-8 channel sealing in this study.

Furthermore, the design of the electrode has a significant influence on the performance of impedance measurement [27]. Most electrode designs for impedance detection are planar electrodes, while few vertical electrodes have been reported. Cheng *et al.* demonstrated that top-bottom electrodes have the highest detection sensitivity (9.18 × 10^−7^ Ω^−1^ (cells/μL)) compared with other electrode designs [28]. However, the limiting factor for top-bottom electrodes may be associated with the difficulty to inspect them due to opacity of most electrodes. Thus, it is not suitable for optical detection when optical detection is required. Hu *et al.* demonstrated the fabrication of three-dimensional (3D) thin-film microelectrode arrays by sputtering Au film on patterned Durimide 7510 film, resulting in trapezoidal side wall electrodes [29]. To generate a uniform electric field along the microchannel height for impedance measurement, the vertical side wall electrodes are preferred rather than the trapezoidal side wall electrodes. Hu *et al.* also reported on the fabrication of continuous and discrete vertical side wall microelectrodes using thin film technology [30,31,32]. The aim of the reported 3D microelectrodes is to improve cell pairing and fusion efficiencies. However, the reported fabrication process of 3D side wall microelectrodes is complicated, especially for discrete vertical side wall microelectrodes. The electroplating method provides an alternative method to fabricate the 3D electrode with a high aspect ratio [24]. To simplify the fabrication process, the electroplating method and thin film technologies are used to fabricate copper electrodes in this paper. As one of the bottom-up manufacturing methods, electroplating is material-saving and a suitable method for our purpose. The aim of the present research is to evaluate the performance of impedance measurement in the proposed microfluidic device. The device has an SU-8 channel and is sealed by direct bonding between SU-8 and PDMS. Additionally, 3D electrodes in the side walls are fabricated for impedance detection and the detection is implemented in the frequency of 1 Hz to 100 kHz by an electrochemical workstation. To investigate the application of the proposed device in the seafood security area, NaCl solution was used in this paper as an electrolyte solution for impedance detection. Different sizes of polystyrene microspheres and different concentrations of polystyrene microspheres mixed in NaCl solution were used to characterize the performance of impedance measurement in the proposed microfluidic device.

## 2. Device Fabrication

### 2.1. Design and Layout

**Figure 1:**
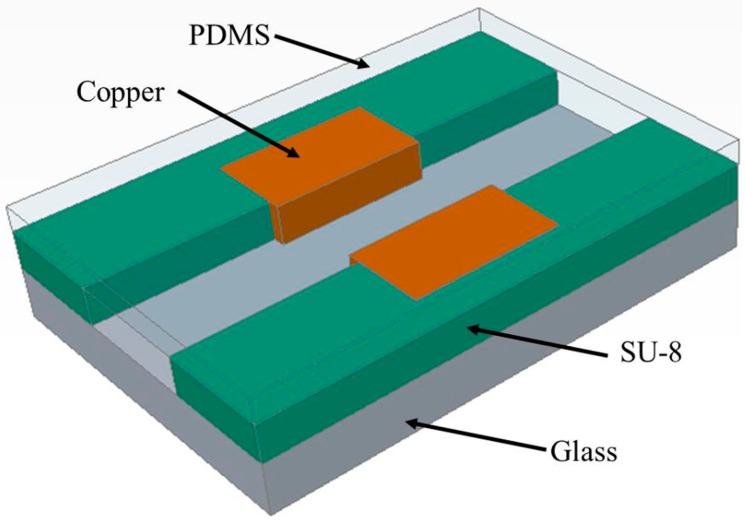
The 3D model of the microfluidic device for impedance measurement.

The device is fabricated on a 3-inch glass wafer which is transparent and low-cost. It contains two parts: the vertical electrodes and the SU-8 microchannel, as shown in Figure 1. It is obvious that the planar electrodes and trapezoidal side wall electrodes would generate a non-uniform electric field. Hence, the results of impedance measurement may be dependent on the exact position of the samples with respect to the electrodes, resulting in a reduced reproducibility [8]. The vertical electrodes fabricated by electroplating were used to generate a uniform electric field. Copper electrodes were used in this paper by taking the advantages of low cost, high conductivity, and the mature processing technology of electroplating. As an epoxy-based photoresist, SU-8 can be patterned flexibly. SU-8 can also be used as the microchannel structures in reported literatures [33]. Due to its optical transparency and inertia to most chemicals, SU-8 has been widely used in biological and chemical areas [34,35]. A flexible PDMS layer is used as a cap to seal the channel since it can deform, leading to contact with the surface it comes into. The width of the channel is designed to be 500 μm and the distance between electrodes is 400 μm to ensure that the electrodes are not covered by the insulating SU-8 photoresist. The length of the channel is 30 mm and the height is 100 μm. The 3D vertical electrodes are 500 μm in width and the thickness is controlled to have a value of 90 μm by the copper electroplating process. Both ends of the microchannel connect two fluid reservoirs that are 3 mm in diameter.

### 2.2. Fabrication of Impedance Sensor

**Figure 2:**
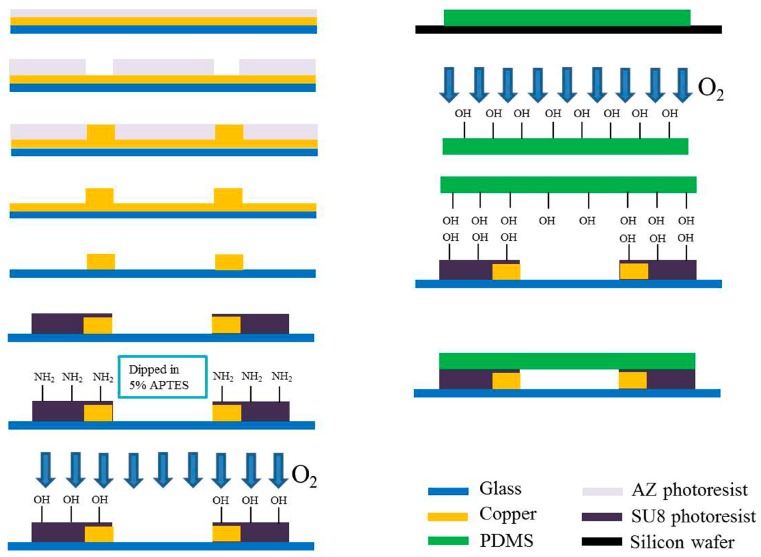
Fabrication process of proposed microfluidic device. The bonding principle of SU-8 with PDMS is based on the modification of Si-containing functionalities on the SU-8 surface.

The detailed fabrication process of the proposed microfluidic device is shown in Figure 2. Firstly, a Ti/Cu film was deposited on a glass wafer as a seed layer for copper electroplating. AZ 4620 (Shipley, Waltham, MA, USA) was coated on the glass wafer at 2000 rpm, after which the wafer was baked for 3 min at 100 °C. After a 30 s exposure, the wafer was developed to form the pattern of electrode and electrical contact. The wafer was subsequently immersed in an activating agent for 40 s before electroplating. After rinsing and drying, the wafer was dipped in a copper electroplating solution with accuracy control of the stir rate and current density for uniform electroplating. The thickness of the electrode controlled to 90 μm was measured by a surface profiler (XP-2, AMBIOS, Santa Cruz, CA, USA). Since the electroplating rate significantly influenced the smoothness and the uniformity of the electroplated electrodes, the current density with significant impact on the electroplating rate was accurately controlled. The etch process should be stopped immediately when the seed layer is completely removed. To remove the moisture before following the coating process, the wafer was baked at 120 °C for 20 min after rinsing. SU-8 photoresist (GM 1075, Gersteltec Sarl, Pully, Switzerland) at 1700 rpm was coated on the wafer to form the structure of microchannel. After photolithography of the SU-8 photoresist, the microchannel was formed with the width slightly larger than the electrode gap to ensure that the electrodes were not covered by the SU-8 photoresist.

### 2.3. Bonding of SU-8 with PDMS

The PDMS (Sylgard 184 from Dow Corning, Midland, MI, USA) which consists of a base polymer and a curing agent was used as the sealing layer. After mixing the two components in a ratio of 10:1 by volume and followed by sufficient de-bubbling of the mixture under vacuum, it was cast in a mold to yield 3-mm-thickness elastomeric sheets. The curing process was performed at 100 °C for 1 h. In order to irreversibly bond the PDMS and SU-8 layer, SU-8 surface silylation was obtained by dipping the SU-8 layer into 5% *v*/*v* 3-APTES solution. The detailed bonding process was according to the report by Vlachopoulou *et al.* [25]. The treated surfaces of SU-8 and PDMS were aligned with respect to each other and brought into contact immediately after plasma activation. To avoid the immediate partial bonding, ethyl alcohol was used as a lubricant during alignment. Complete bonding was achieved by placing them on a hot-plate for 30 min at 100 °C with a 0.5 kg weight placed on top of them to ensure tight contact.

**Figure 3:**
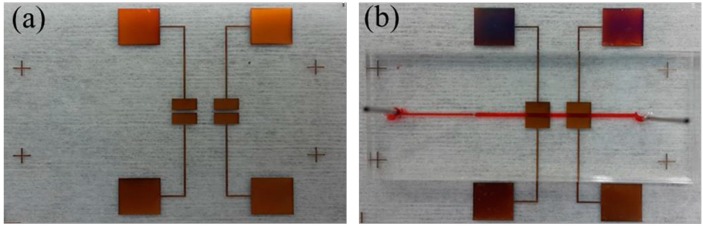
Fabricated device before (**a**) and after having been sealed by PDMS (**b**).

The device before and after having been sealed by the PDMS layer is shown in Figure 3. The spacing of the electrodes after fabrication was measured to be 398.75 μm, as the nominal value was 400 μm. The sealing effect was evaluated by injecting dye solution into the channel for the detection of leakage. As shown in Figure 3b, no leakage was found at flow rates above 2 mL/min. Bond strength of around 1.5 MPa was measured as the maximum endurable strength between the PDMS and SU-8 [36]. In addition, the possibility of clogging the channel due to uncured SU-8 was also avoided as no viscous material existed during the bonding process.

## 3. Experiment

### 3.1. Bonding Test

To investigate the bonding mechanism between the SU-8 and PDMS, the contact angle and Fourier Transform Infrared (FTIR) spectroscopy measurements were used for the characterization of SU-8 modification. SU-8 GM1075 was spun onto a glass wafer with the spin rate of 1700 rpm and followed by pre-bake for 2 min at 120 °C. After a 11 s exposure, the wafer was post-baked 30 min at 95 °C. The glass wafer coated with SU-8 was cut into pieces with dimensions of 80 mm × 80 mm by dicing saw (ADT 7100, Advanced Dicing Technology, Yokneam, Israel). These pieces were used for the characterization of SU-8 modification by contact angle measurement. Part of these pieces were treated by 3-APTES, resulting in surface silylation, and then heated for 20 min at 80 °C on a hot-plate. Part of the modified pieces were further treated by oxygen plasma with a processing time of 20 s. To obtain FTIR spectroscopy, a thin layer of SU-8 was spun-coated on Au substrate. The coating processes were the same as the treatment of glass pieces coated with SU-8 as mentioned above.

### 3.2. Preparation for Impedance Measurement

NaCl solutions were prepared with the concentrations of 0.6, 1 and 3 M. By injecting the NaCl solution into the device, the impedances of different concentrations of NaCl solutions were measured by the Zennium electrochemical workstation. The impedances of NaCl solutions mixed with polystyrene microspheres were also measured and analyzed. Solution with a concentration of 5% w/v polystyrene microspheres (purchased from Spherotech Inc., Lake Forest, IL, USA) suspension in 0.016 M PBS, pH 7.4 with 0.02% Sodium Azide and 0.05% bovine serum albumin (BSA) was diluted in 0.6 M NaCl solution with volume ratios of 1:100, 1:200, 1:400, 1:800, and 1:1600. Additionally, in order to investigate the ability of distinguishing different sizes of microspheres, solutions containing different sizes of polystyrene microspheres were also injected into the microchannel and analyzed. Polystyrene microspheres with dimensions of 2.78 μm, 3.43 μm and 5.98 μm were diluted in 0.6 M NaCl solution with the same volume ratio of 1:200.

## 4. Results and Discussion

### 4.1. Contact Angle and FTIR Spectra Characterization of Modified SU-8 Layer

**Figure 4:**
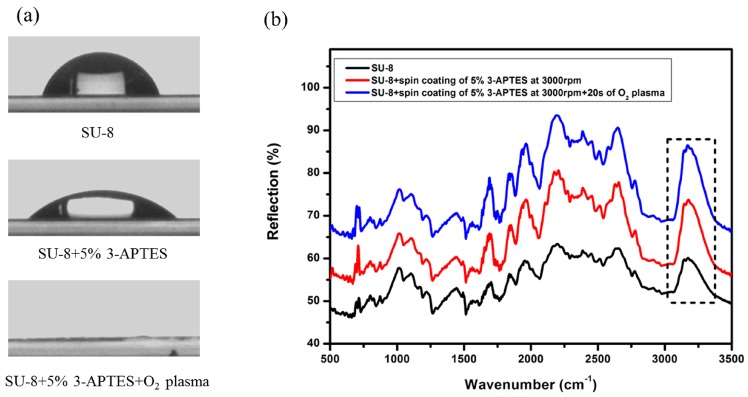
The contact angle varies after surface modification (**a**) and FTIR reflection spectra of SU-8 before and after treatment with APTES and the peak assignment (**b**). Peaks at 3172 cm^−1^ contribute to -OH group at different processing stages.

The bonding process of PDMS with SU-8 was characterized by contact angle measurement and FTIR spectroscopy, and the results are shown in Figure 4. Firstly, the contact angle of the original SU-8 layer was measured and it exhibited a contact angle of 69.2°. Subsequently, the SU-8 with the incorporation of Si-containing functionalities on surface by 3-APTES treatment was verified by contact angle measurement. The measured value was 41.6° which indicates that the functionalized surface became more hydrophilic. This result is close to the presence of the amine (-NH_2_)-terminated layer on the PMMA surface [37,38]. After the oxygen plasma treatment of amine-functionalized SU-8, the measured contact angle was 0°, which indicates that the surface became completely hydrophilic. The presence of Si-containing functionalities on the SU-8 surface was further characterized by FTIR spectroscopy, and the spectra are shown in Figure 4b. Since SU-8 is a mixture composed mainly of novolac phenol epoxy resin mixed in Gamma Butyrolacton and photoinitiator, the composition of SU-8 is complex and it is difficult to distinguish peaks. However, it is clear that the content of the hydroxyl group increases after the two steps of surface modification, which is the main contribution to the irreversible bonding between PDMS and SU-8 [25].

### 4.2. Impedance Detection of NaCl Solution

**Figure 5:**
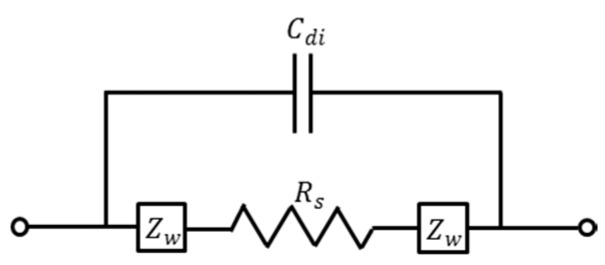
Circuit model of impedance measurement for electrolytic solution.

To understand the impedance measurement of electrolytic solution, a fairly simple equivalent circuit can be used for electrodes in an electrolytic solution, as shown in Figure 5 [39,40]. In this model, *C_di_* accounts for the dielectric capacitance and it contains dielectric contributions from all the materials surrounding the electrodes, $R_{s}$ is the bulk solution resistance which represents charge transfer resistance across the bulk solution, and $Z_{W}$ is the interfacial impedance (also called Warburg impedance) which represent the change in the ionic gradient at the interface. The interfacial impedance $Z_{W}$ is given by the following expression:

(1) $$Z_{i}=\frac{1}{{\left(j\omega \right)}^{n_{i}}B_{i}}$$

where $j=\sqrt{\left(-1\right)}$, n and B are parameters which are dependent of the properties of the electrolytic solution and the electrodes.

**Figure 6:**
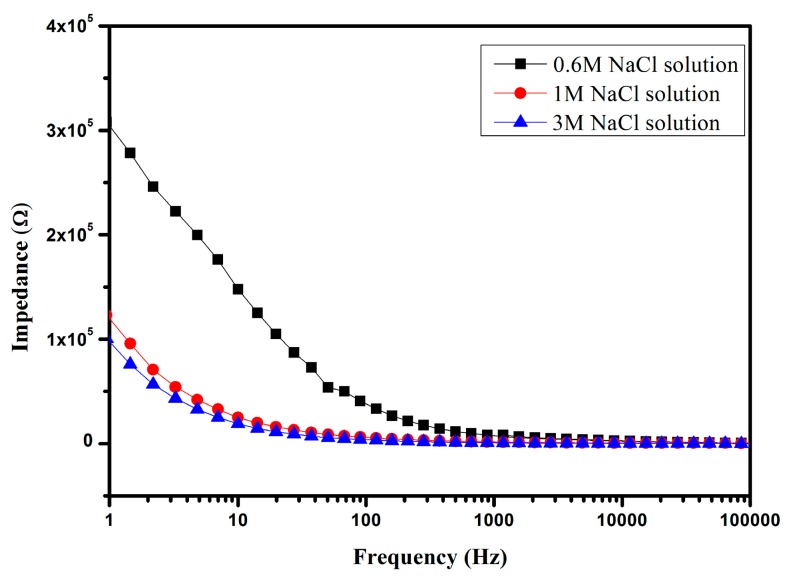
Values of impedances of measured NaCl solutions as a function of frequency.

The solutions were sufficiently mixed before being injected into the microchannel. It should be insured that there are no bubbles in the microchannel during the measurement, since bubbles may result in significant interference. The flow rate was controlled to be 100 μL/min by the syringe pump. After the solution had completely developed and reached a steady state, the impedance measurement was started. The impedance signals of NaCl solutions with concentrations of 0.6, 1 and 3 M were obtained, as illustrated in Figure 6a. It is obvious that the values of impedance show exponential damping with the frequency increasing in the range of 1 Hz to 100 kHz. The variation trend of impedance agrees with the literature observations [8,41,42]. The solutions with different concentrations have different values of impedance, for instance 0.6 M NaCl has the maximum impedance value of 307.96 kΩ at 1 Hz while the 3 M NaCl solution has the minimum impedance value of 100.44 kΩ under the same frequency. The impedance of the solution with higher concentration decays more slowly as compared with other solutions. The difference between the impedances of solutions with different concentrations decreases as the frequency increases. The impedances are approximately equal when the frequency is higher than 10 kHz. It is clear that the changing trends of the measured impedances of solutions with different concentrations are in accordance with the corresponding conductivities at low frequency, as solutions with higher conductivity have relatively lower impedances.

### 4.3. Impedance Detection of Microspheres

**Figure 7:**
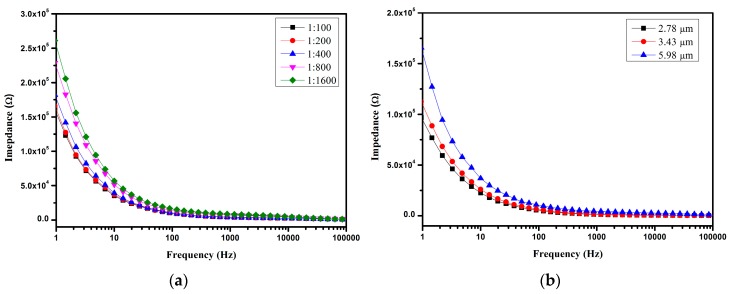
Impedance measurements of different volume ratios of solutions containing 5.98 μm polystyrene microspheres (**a**) and solutions containing different diameters of polystyrene microspheres (**b**). Different diameters of polystyrene microspheres were diluted in 0.6 M NaCl solution with the same volume ratio of 1:200.

A significant difference was found between different concentrations of microspheres, as shown in Figure 7a. This may be due to the conductivity of the NaCl solution being improved when polystyrene microspheres were added. With the increase in the concentration of polystyrene microspheres, the conductivity of the mixture solution also increased. This may due to the dielectric relaxation in polystyrene suspensions related to the polarization of the electric double layer [43,44]. Further validation should be performed in the future. It can therefore be seen that the proposed device has the ability to distinguish different concentrations of polystyrene microsphere solutions, supported by the results as shown in Figure 7b. This indicates that solutions containing different sizes of microspheres have different impedance spectroscopies. The solution containing a larger-diameter microsphere resulted in a higher impedance. It can be explained that the conductivity of spheres $\sigma_{P}$ is governed by the bulk conductivity and the surface conductivity. Its relationship with the surface and the conductivity of spheres $\sigma_{P}$ is expressed as follows [45,46]:

(2) $$\sigma_{P}=\sigma_{P-Bulk}+\frac{2K_{S}}{r}$$

where $\sigma_{P-Bulk}$ is the bulk conductivity, $K_{S}$ is the surface conductance which composes the contributions of the stern layer and diffuse layer formed around the spheres, and *r* is the radius of the spheres. According to Equation (1), the conductivity of spheres decreases with the increase in the radius of the sphere; thus, the solution containing larger-radius spheres has a higher impedance. It is clear that the proposed microfluidic device is suitable for impedance detection of solution with different concentrations of spheres and also for the distinction of spheres with different sizes.

## 5. Conclusions 

The results show that the proposed microfluidic device for impedance measurement is feasible and is applicable in the microfluidic area. At a frequency lower than 10 kHz, the magnitude of the impedance decreases as the frequency increases. The change in the rate of impedance amplitude is considered to be related to the solution concentration, and the impedance of a solution with a higher concentration has a comparatively slower rate. The proposed microfluidic device is suitable for the impedance detection of different solution concentrations and has the ability to detect different sizes of microspheres. The design of the electrode should be further studied as the optimized design of the electrode provides improved sensitivities.
